# Characterization of the complete chloroplast genome of *Puccinellia distans*

**DOI:** 10.1080/23802359.2021.1882899

**Published:** 2021-03-11

**Authors:** Yan Zhang, Minjie Liu, Yan Qin, Wenhui Liu, Xiaoxing Wei

**Affiliations:** Academy of Animal and Veterinary Sciences, Qinghai University, Xining, Qinghai, PR China

**Keywords:** *Puccinellia distans*, chloroplast genome, phylogenetic relationship

## Abstract

*Puccinellia distans* is a perennial gramineous plant with the characteristics of drought and salt tolerance. It is a special pioneer plant for saline-alkali land improvement and is increasingly used for ecological restoration of saline-alkali grassland. However, the evolutionary relationship of *P. distans* is limited in study. In this study, the complete chloroplast genome sequence of *P. distans* was evaluated. The complete chloroplast genome of *P. distans* was 135,647 bp in length, containing a pair of inverted repeated (IR) regions (21,444 bp) that are separated by a large single-copy (LSC) region of 800,15 bp, and a small single-copy (SSC) region of 12,744 bp. A total of 129 functional genes were annotated, including 83 protein-coding genes (mRNA), 38 tRNA genes, and 8 rRNA genes. The phylogenetic relationships of 12 species indicated that *P. distans* was closely related to *P. muttalliana*. This complete chloroplast genome will provide a theoretical basis for species identification and biological research.

*Puccinellia distans* is a perennial, monocotyledonous C3 plant of Poaceae family, mainly distributed in the Northeast and Northwest of China (Elisabetta et al. 2019). It is an excellent forage grass with the characteristics of early turning green, high nutritional value, and good palatability (Sun 2014). In addition, *P. distans* is a special pioneer plant for saline-alkali soil improvement because of its excellent drought and salt tolerance (Zhang et al. 2013). It is gradually used for ecological restoration (Peng et al. 2004). However, the evolutionary relationship of *P. distans* is limited. In this study, the complete chloroplast genome of *P. distans* was sequenced on the Illumina NovaSeq platform (Genbank accession number: MW044608), which would provide a theoretical basis for species identification and biological research in future study.

The fresh leave samples of *P. distan*s were collected in experiment station of perennial forage in Xihai Town, Haibei Prefecture, Qinghai, China (36°49′297″N, 101°45′266″E). The voucher specimen was kept in Herbarium of Low-temperature library of Germplasm resources in Qinghai Academy of Animal and Veterinary Sciences (09-129, contact person and email are Xiaoxing Wei, wuiko@163.com). The total genomic DNA of *P. distan*s was extracted from the fresh leaves with a modified CTAB method (Li et al. 2018). One library was constructed using PCR amplification. The template size is 300 bp. The genome sequencing was performed with an Illumina NovaSeq platform (Genepioneer Biotechnologies, Nanjing, China). The trimmed reads were mainly assembled using SPAdes (Bankevich et al. 2012). The assembled genome was annotated using Cp GAVAS (Liu et al. 2012).

The complete chloroplast genome of *P. distans* was 135,647 bp in length, containing a pair of inverted repeated (IR) regions (21,444 bp) that are separated by a large single copy (LSC) region of 800,15 bp, and a small single copy (SSC) region of 12,744 bp. The GC content of the whole chloroplast genome was 38.32%. A total of 129 functional genes were annotated, including 83 protein-coding genes (mRNA), 38 tRNA genes, and 8 rRNA genes. The protein-coding genes, tRNA genes, and rRNA genes account for 64.34%, 29.46%, and 6.20% of all annotated genes, respectively.

To reveal the phylogenetic position of *P. distans*, a maximum likelihood (ML) phylogenetic tree (Figure 1) was constructed with MEGA version 7 (Huang et al. 2020) using the coding sequences of 12 species. All the 12 species are Gramineae and belong to different genera. The results showed that *P. distans* was closely related to *P. muttalliana*. This study will contribute to its future breeding and biological research.

**Figure 1.**
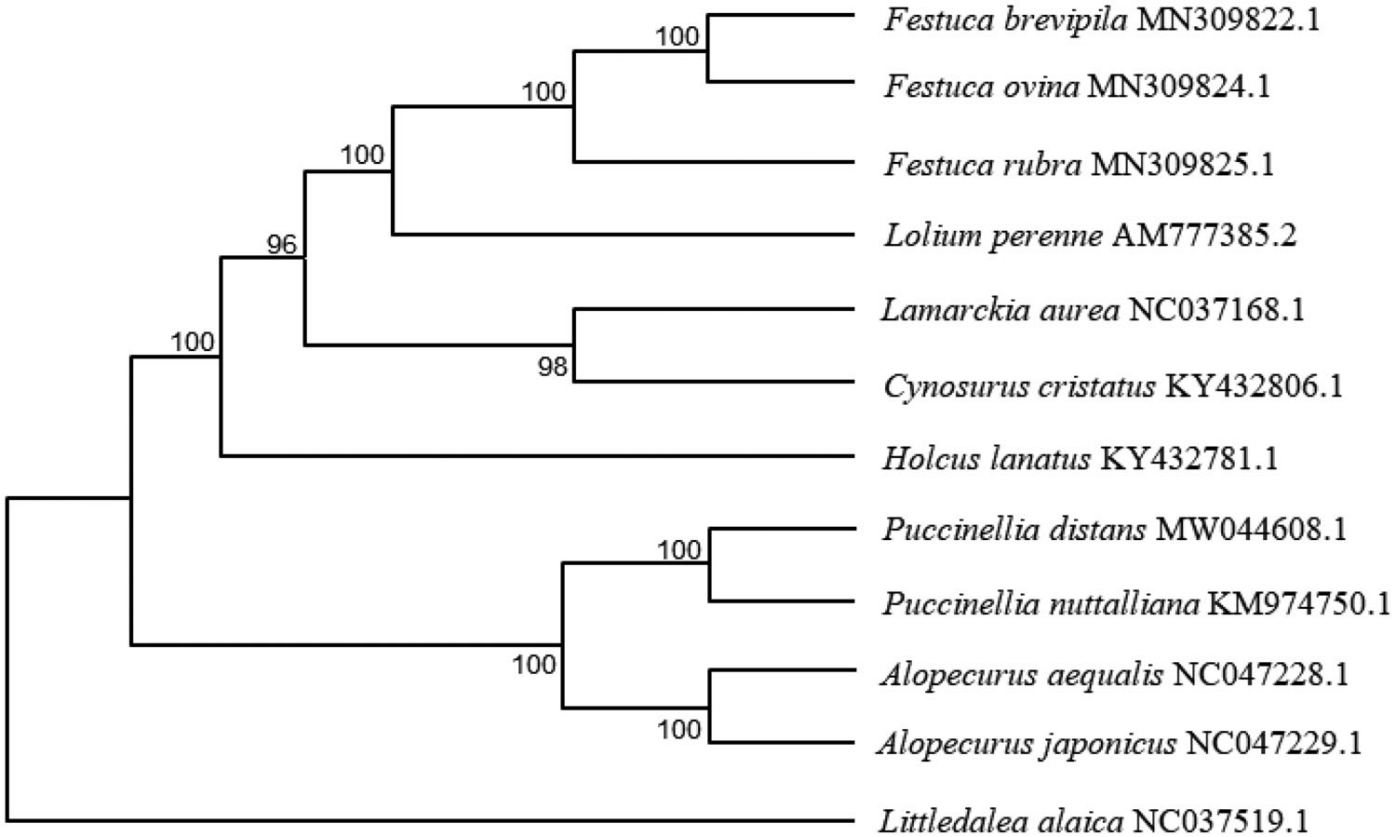
Phylogenetic relationships of 12 species based on complete chloroplast genome using the maximum likelihood methods. The bootstrap values were based on 1000 replicates and are shown next to the branches.

## Data Availability

The genome sequence data that support the findings of this study are openly available in GenBank of NCBI at (https://www.ncbi.nlm.nih.gov/) under the accession no.MW044608. The associated Bio-Project, SRA, and Bio-Sample numbers are PRJNA680621, SRR13375866, and SAMN16898198 respectively.
